# mSphere of Influence: Redefining an Influenza Virus—How Different Are Influenza Viruses and Extracellular Vesicles?

**DOI:** 10.1128/mSphere.00563-19

**Published:** 2019-09-04

**Authors:** Seema S. Lakdawala

**Affiliations:** a Department of Microbiology and Molecular Genetics, University of Pittsburgh School of Medicine, Pittsburgh, Pennsylvania, USA

## Abstract

Seema Lakdawala works in the field of influenza virology. In this mSphere of Influence article, she reflects on how the article “Conserved and host-specific features of influenza virion architecture” by Edward C. Hutchinson et al. (Nat Commun 5:4816, 2014, https://doi.org/10.1038/ncomms5816) made an impact on her thinking about what constitutes an infectious virus and how influenza viruses can spread within and among hosts.

## Abstract (precis)

Seema Lakdawala works in the field of influenza virology. In this mSphere of Influence article, she reflects on how the article “Conserved and host-specific features of influenza virion architecture” by Edward C.

## COMMENTARY

An image of influenza viruses is clear to most biologists—a circular blob speckled with viral glycoproteins: hemagglutinin (HA) and neuraminidase (NA). In “Conserved and host-specific features of influenza virion architecture” (1), the authors reexamined the proteome of influenza virions grown in traditional cell culture or chicken eggs using liquid chromatography and tandem mass spectroscopy (LC-MS/MS). In addition to the viral proteome, a large number of host proteins were identified in influenza virions, which are similar to protein markers of extracellular microvesicles. Importantly, they observed differences in the protein composition of influenza viruses generated in chicken eggs compared to human cells. This observation, in the context of other recent publications demonstrating that viral RNA can be released within extracellular vesicles (2, 3), has reshaped my own thinking about what constitutes an infectious virus and how influenza viruses can spread within an individual host and between different host populations.

Influenza viruses bud from the plasma membrane of a host cell, similar to some extracellular microvesicle populations (4). Hutchinson and colleagues performed label-free absolute quantification of viral and host proteins in purified virion preparations. Their purification process is critical, because they used heme absorption to differentiate influenza virions from host extracellular microvesicles that lacked the HA protein. HA is known to agglutinate red blood cells, and heme absorption will enrich HA-containing viruses onto large red blood cells that can be pelleted out of solution. This processing step is critical because some extracellular microvesicles are similar in size to influenza virions (∼100 nm) (4). Previous studies exploring the protein content of influenza viruses may have been biased with host extracellular vesicles contaminating virus cultures; thus, this new methodology to separate HA-containing virions from microvesicles provides a clearer picture of the influenza viral proteome.

Extracellular microvesicles transport protein or nucleic acid signals throughout the body, which at a basic level is the same function as viruses. Research on extracellular microvesicles during viral infections has suggested a lack of delineation between extracellular microvesicles and virion populations, since they both contain similar proteins and nucleic acids (5). The heterogenous population of viruses and extracellular vesicles may be an important component of viral pathogenies and spread. In addition, recent data from my group and others have demonstrated that the viral RNA inside influenza virions is not uniformly bound to the viral nucleoprotein and that regions of the viral RNA are flexible (6–9). Taken together with the data presented by Hutchinson et al., it is likely that some of the host components identified through virion LC-MS/MS may also be bound to viral RNA. The association of host proteins with viral RNA may impact the replication capacity of certain segments in subsequent infections. Additional research is needed to answer many open questions that arise from these findings. Does the viral proteome differ based on cell type and virus strain? Can changes in the viral proteome impact infectivity of influenza virions? How are transmission and pathogenesis of influenza viruses impacted by the incorporation of host proteins?

A graphical coloring page based on the data presented by Hutchinson et al. (1) is freely available from Art Goes Viral (https://www.gla.ac.uk/media/media_531204_en.pdf) or in reference 10 and is a visual representation of how the architecture of an influenza virion has changed.

## References

[1] Hutchinson EC, Charles PD, Hester SS, Thomas B, Trudgian D, Martinez-Alonso M, Fodor E 2014 Conserved and host-specific features of influenza virion architecture. Nat Commun 5:4816. doi:10.1038/ncomms5816. 25226414 PMC4167602

[2] Altan-Bonnet N 2016 Extracellular vesicles are the Trojan horses of viral infection. Curr Opin Microbiol 32:77–81. doi:10.1016/j.mib.2016.05.004. 27232382 PMC4983493

[3] Longatti A, Boyd B, Chisari FV 2015 Virion-independent transfer of replication-competent hepatitis C virus RNA between permissive cells. J Virol 89:2956–2961. doi:10.1128/JVI.02721-14. 25505060 PMC4325714

[4] Tkach M, Thery C 2016 Communication by extracellular vesicles: where we are and where we need to go. Cell 164:1226–1232. doi:10.1016/j.cell.2016.01.043. 26967288

[5] Nolte-'t Hoen E, Cremer T, Gallo RC, Margolis LB 2016 Extracellular vesicles and viruses: are they close relatives? Proc Natl Acad Sci U S A 113:9155–9161. doi:10.1073/pnas.1605146113. 27432966 PMC4995926

[6] Lee N, Le Sage V, Nanni AV, Snyder DJ, Cooper VS, Lakdawala SS 2017 Genome-wide analysis of influenza viral RNA and nucleoprotein association. Nucleic Acids Res 45:8968–8977. doi:10.1093/nar/gkx584. 28911100 PMC5587783

[7] Le Sage V, Nanni AV, Bhagwat AR, Snyder DJ, Cooper VS, Lakdawala SS, Lee N 2018 Non-uniform and non-random binding of nucleoprotein to influenza A and B viral RNA. Viruses 10:E522. doi:10.3390/v10100522. 30257455 PMC6213415

[8] Dadonaite B, Gilbertson B, Knight ML, Trifkovic S, Rockman S, Laederach A, Brown LE, Fodor E, Bauer DLV 2019 The structure of the influenza A virus genome. Nat Microbiol doi:10.1038/s41564-019-0513-7. PMC7191640 31332385

[9] Williams GD, Townsend D, Wylie KM, Kim PJ, Amarasinghe GK, Kutluay SB, Boon A 2018 Nucleotide resolution mapping of influenza A virus nucleoprotein-RNA interactions reveals RNA features required for replication. Nat Commun 9:465. doi:10.1038/s41467-018-02886-w. 29386621 PMC5792457

[10] Lakdawala SS, Nair N, Hutchinson E 2019 Educational material about influenza viruses. Viruses 11:231. doi:10.3390/v11030231. PMC6466267 30866478

